# Task-Induced Deactivation from Rest Extends beyond the Default Mode Brain Network

**DOI:** 10.1371/journal.pone.0022964

**Published:** 2011-07-29

**Authors:** Ben J. Harrison, Jesus Pujol, Oren Contreras-Rodríguez, Carles Soriano-Mas, Marina López-Solà, Joan Deus, Hector Ortiz, Laura Blanco-Hinojo, Pino Alonso, Rosa Hernández-Ribas, Narcís Cardoner, José M. Menchón

**Affiliations:** 1 Institut d'Alta Tecnologia-PRBB, CRC Mar, Hospital del Mar, Barcelona, Spain; 2 Department of Psychiatry, Melbourne Neuropsychiatry Centre, The University of Melbourne and Melbourne Health, Melbourne, Australia; 3 Fundació Institut Municipal d'Investigació Mèdica (IMIM), Barcelona, Spain; 4 Department of Clinical and Health Psychology, Autonomous University of Barcelona, Barcelona, Spain; 5 Neuroscience Group-IDIBELL, Department of Psychiatry, Bellvitge University Hospital-ICS, University of Barcelona, CIBERSAM, Barcelona, Spain; University of Granada, Spain

## Abstract

Activity decreases, or deactivations, of midline and parietal cortical brain regions are routinely observed in human functional neuroimaging studies that compare periods of task-based cognitive performance with passive states, such as rest. It is now widely held that such task-induced deactivations index a highly organized ‘default-mode network’ (DMN): a large-scale brain system whose discovery has had broad implications in the study of human brain function and behavior. In this work, we show that common task-induced deactivations from rest also occur outside of the DMN as a function of increased task demand. Fifty healthy adult subjects performed two distinct functional magnetic resonance imaging tasks that were designed to reliably map deactivations from a resting baseline. As primary findings, increases in task demand consistently modulated the regional anatomy of DMN deactivation. At high levels of task demand, robust deactivation was observed in non-DMN regions, most notably, the posterior insular cortex. Deactivation of this region was directly implicated in a performance-based analysis of experienced task difficulty. Together, these findings suggest that task-induced deactivations from rest are not limited to the DMN and extend to brain regions typically associated with integrative sensory and interoceptive processes.

## Introduction

While the primary use of functional magnetic resonance imaging (fMRI) remains in the context of brain activation studies, the relevance of functional decreases or deactivations of brain activity has also attracted considerable recent interest. This interest centers on the observation that a common set of brain regions show prominent functional decreases from baseline to task epochs and most specifically when comparing resting baselines to periods of cognitive task performance. Raichle et al., [1] proposed that this deactivation pattern may represent an operational “default-mode of brain function”, whereby such comparisons unmasked a pattern of higher ongoing regional activity at rest that is attenuated during goal-directed behavior. This characteristic deactivated anatomy, or “default-mode network” (DMN), has been replicated in numerous subsequent studies and primarily involves the posterior cingulate cortex, the medial frontal cortex and the angular gyrus region of the inferior parietal cortex [2], [3], [4]; reviewed in [5]. Recent studies mapping the functional connectivity of these regions under continuous resting-state fMRI conditions further implicate the hippocampus, inferior temporal cortex and precuneus as part of the DMN [5].

There is growing recognition that task-induced deactivations of the DMN may represent an important functional correlate of goal-directed behavior– having been directly linked with task outcome measures in several recent studies [6], [7], [8], [9], [10], [11], [12]. One current view is that these deactivations may correspond to self-focused spontaneous cognition, or “mind-wandering”, that is common during wakeful resting conditions, but that must be tuned down during task-focused behavior [13], [14], [15], [16]. These ideas have been advanced by recent activation studies that have linked specific DMN regions to distinct self-related cognitive domains, such as autobiographical memory, prospection, and moral-social reasoning [15], [17], [18], [19]. By comparison, fewer studies have addressed the potential specificity of DMN regions with respect to task-induced deactivations, favoring the view that such deactivations are mostly common or task-independent.

Despite being broadly consistent across studies comparing different ‘high level’ cognitive tasks with resting baselines [2], [3], a close inspection of past findings also suggests that important variability exists in the deactivation of DMN regions. Differences in the precise anatomy of deactivated regions can be consistently observed between independent studies, particularly with regards to the posterior cingulate and medial frontal cortex, as can the finding of additional deactivations in brain regions that appear outside of the DMN [13], [20], [21], [22]. For example, in a recent study, Mayer et al., [21] used a factorial design to independently modulate task demands on working memory and visual attention compared to a resting baseline. Although overall both tasks robustly deactivated DMN regions, the regions which showed a common and additive effect of task demand only marginally encompassed the DMN. Other brain regions, including the posterior insular cortex, were more consistently responsive to changes in task demand.

The possibility that additional “non-DMN” regions may consistently show task-induced deactivation from a resting baseline has yet to be firmly acknowledged on the basis of existing studies despite being apparent across a number of them [10], [13], [20], [21]. Nevertheless, there is reason to suspect that such deactivations may also represent important functional correlates of task-focused behavior. The posterior insular cortex, for instance, is broadly activated in functional imaging studies involving somatosensory, nociceptive and interoceptive stimulation [23], consistent with its description as a multimodal integration area [24], or “insular association cortex” [25]. Interestingly, deactivation of this region has been associated with reduced awareness of painful stimulation during Stroop task performance, suggesting that cognitive task demand also has a potent modulatory influence on the functionality of this region and corresponding subjective states [26].

The goal of the present study was to further investigate the anatomical specificity of task-induced deactivations from a resting baseline, particularly in response to common increases in task demand. Based on existing evidence, we made the following predictions: *i)* that the anatomy of deactivation would be clearly modulated by increases in task demand compared to rest, and *ii)* that such modulation would extend to non-DMN regions, namely the posterior insular cortex, at high task demand levels. While past studies have investigated the specificity of DMN deactivations to multiple task contexts in the same subjects, the modulatory effect of task demand on the anatomy of deactivations has been less clearly established. To test our predictions, we assessed a large sample of healthy subjects with two well known but distinct tasks–an emotional face matching task and cognitive (Stroop) interference task– that were modified in order to reliably map deactivations from a resting baseline. The tasks were considered distinct in that they putatively engage different cognitive modalities and lead to functional activation patterns that are mostly non-overlapping, unlike tasks used in other recent work [21], [22]. Thus, with this approach, we reasoned that the existence of common versus specific effects of task demand on the anatomy of deactivations might be more readily apparent.

## Materials and Methods

### Subjects

Fifty healthy adult subjects were included in this study (25 female; mean age ± SD = 31.8±8.8 years; range 18–52). None had a personal history of neurological or psychiatric illness and all had normal or corrected-to-normal vision. Subjects gave written, informed consent to participate in the study, which was approved by the Research and Ethics Committee of the Institut Municipal d'Investigació Mèdica (IMIM)-Hospital del Mar (Barcelona, Spain).

### Experimental tasks

To test our study predictions, we chose two independent blocked-design tasks that have been widely studied with functional neuroimaging and modified them in order to reliably map task-induced deactivations from a resting baseline. To do so, each task block–as detailed below–was interleaved with 12 second (s) periods of rest-fixation. To ensure that the total duration of the resting condition was equivalent to the total duration of each specific task condition, both tasks commenced with an initial rest-fixation period of 32 s.

#### Task I

This task represented a modified version of the emotional face matching paradigm originally reported by Hariri et al., [27] and as recently described in Pujol et al., [28]. Although this particular task was additionally chosen to satisfy the aims a larger clinical neuroimaging protocol [28], [29], it was also purposefully modified from its original format in order to study corresponding deactivations of the DMN. Briefly, subjects responded to 30 s pseudo-randomized blocks of shape matching (‘lower task demand’) and emotional face matching (‘higher task demand’) trials, totaling 12 performance blocks. Each block included 6 different stimulus trials (stimulus interval, 5 s). During each 5 s shape trial, subjects were presented with either an oval or circle target shape (centrally located at the top a white screen) and two probe shapes (bottom left and right of screen) and were instructed to match “as accurately as possible” the probe to the target shape by pressing left or right button responses with the second and third fingers of their right hand. During emotional face trials, subjects were presented with a target emotional face (top center) and two probe faces (bottom left and right) in an analogous configuration and were instructed to match “as accurately as possible” the probe expressing the same emotion to the target by button response. The target face was either happy or fearful, and the probe faces included two out of three possible emotional faces (happy, fearful and angry). For our purposes, emotional face matching corresponded to a combined assessment of happy and fearful emotion trial types. The paradigm ran for a total duration of nine minutes.

#### Task II

This task represented modified version of the traditional Stroop color-word interference task, as described in Harrison et al., [17]. Briefly, subjects responded to 30 s blocks of congruent (‘lower task demand’) and incongruent (‘higher task demand’) color-word matching trials, totaling 8 performance blocks. During congruent trials, the stimulus ‘XXXX’ was centered on a black screen in either one of three colors: red, green or blue. Correct responses were mapped to the following target stimuli: ‘RED’, ‘GREEN’ or ‘BLUE’, located below the cue stimulus and displayed in congruent ink color. The location of the targets (left, middle, right) corresponded to specific hand-held button device responses. During incongruent trials, the same stimulus configuration was presented, however, the cue stimulus was instead one of the same three words presented in incongruent ink color. There was no inter-stimulus interval between consecutive stimulus presentations. Instead, new stimuli appeared at a pace determined by each subjects' rate of responding. Subjects were instructed to match the color of the cue stimulus with the corresponding target word stimulus “as accurately *and* quickly as possible”. To avoid motion, they were instructed not to vocalize their response during the task trials. Subjects responded with an approximate response frequency of 1 Hertz (range 2.1 to 0.70 Hz), or 1 response every second. The paradigm ran for a total duration of six minutes.

Both imaging tasks were programmed using Presentation software (http://www.neurobs.com/) and presented to subjects using MRI-compatible high-resolution goggles and headphones (VisuaStim Digital System, Resonance Technology Inc., Northridge, CA). Subjects' reaction time (RT) and error responses were registered online using a hand-held (optical fiber) response device (Nordic NeuroLab, Bergen, Norway). Task performance was assessed behaviorally in terms of mean RT and percentage omission (misses) and commission error responses (incorrect responses) to the task conditions and was analyzed by one-way repeated measures analysis variance (ANOVA) in PASW Statistics 18.0 (SPSS Inc., Chicago, IL).

### Imaging acquisition & preprocessing

A 1.5 T Signa Excite system (General Electric, Milwaukee, WI, USA) equipped with an eight-channel phased-array head coil and single-shot echoplanar imaging (EPI) software was used. Functional sequences consisted of gradient recalled acquisition in the steady state (time of repetition [TR], 2000 ms; time of echo [TE], 50 ms; pulse angle, 90°) within a field of view of 24 cm, with a 64×64-pixel matrix, and with a slice thickness of 4 mm (inter-slice gap, 1 mm). Twenty-two interleaved slices, parallel to the anterior-posterior commissure (AC-PC) line, were acquired to cover the whole-brain for all functional sequences.

Imaging data were transferred and processed on a Microsoft Windows platform running MATLAB version 7 (The MathWorks Inc, Natick, Mass). Image preprocessing was performed with Statistical Parametric Mapping software (SPM5; Wellcome Trust Centre for Neuroimaging, UK) and involved motion correction, spatial normalization and smoothing using a Gaussian filter (full-width, half-maximum, 8 mm). Motion correction was performed by aligning (within-subject) each time-series to the first image volume using a least-squares minimization and a 6-parameter (rigid body) spatial transformation. For all subjects, translation and rotation estimates (x, y, z) were well below 2 mm and 2°, respectively. Data were normalized to the standard SPM-EPI template and resliced to 2 mm isotropic resolution in Montreal Neurological Institute (MNI) space. All image sequences were routinely inspected for potential normalization artifacts.

### Imaging analysis

For each subject, primary task regressors were created by specifying the onset and duration of each task block, followed by convolution with a canonical hemodynamic response function and its temporal derivative. For task I, primary task regressors included the shape matching, emotional face matching and rest-fixation blocks. For task II, primary task regressors included the congruent color-word matching, incongruent color-word matching, and rest-fixation blocks. Maximum likelihood parameter estimates were calculated at each voxel using the general linear model and an AR(1) model of serial correlations. The model additionally included drift terms up to 1/128 Hz to remove low-frequency components, and global confounds were removed using global normalization. SPM contrast images were generated for each subject, as detailed below, to be carried forward to the group level using the summary statistics approach to random effects analysis.

#### Task I

Based on recent findings [30] and our previous experience with the emotional face matching task [28], it was expected that DMN regions would be broadly deactivated in association with the higher demand face matching condition, but only marginally deactivated by the simple sensorimotor demands of the shape matching condition. We focused on three main experimental contrasts to assess task-induced deactivation: *i)* **rest-shapes**, rest-fixation *minus* shape matching blocks, or deactivation during the lower demand task condition; *ii)* **rest-faces**, rest-fixation *minus* face matching blocks, or deactivation during the higher demand task condition; and *iii)* **shapes-faces**, shape matching *minus* face matching blocks, or deactivation specific to the higher demand task condition. Contrast images corresponding to these main task effects were included in a factorial model random-effects analysis (SPM voxel-level family-wise error rate corrected; *P* **_FWE_** *<*0.05, K_E_, ≥10 voxels). Importantly, this model allowed the results of the latter **shapes-faces** contrast to be exclusively masked by the contrast ‘shape matching *minus* rest-fixation’ in order to remove any regional activations associated with the performance of the shape matching blocks. In other words, this third contrast isolated only brain regions that deactivated specifically in response to the higher demand face matching blocks.

#### Task II

Based on our recent experience with this self-paced version of the task [17], we expected DMN regions to be robustly deactivated in association with both trial types such that, in this instance, the lower demand task condition would be sufficiently challenging to broadly deactivate DMN regions. We focused on three main experimental contrasts to assess task-induced deactivation: *i)* **rest-con**, rest-fixation *minus* congruent Stroop blocks, or deactivation during the lower demand task condition; *ii)* **rest-incon**, rest-fixation *minus* incongruent Stroop blocks, or deactivation during the higher demand task condition; and *iii)* **con-incon**, congruent *minus* incongruent Stroop blocks, or deactivation specific to high demand task condition. Contrast images corresponding to these main task effects were also included in a factorial model random-effects analysis (SPM voxel-level family-wise error rate corrected; *P* **_FWE_** *<*0.05, K_E_, ≥10 voxels). This model additionally allowed the results of the **con-incon** contrast to be exclusively masked by the contrast ‘congruent *minus* rest-fixation’ in order to remove any regional activations associated with the performance of the congruent Stroop blocks. In other words, this third contrast isolated only brain regions that deactivated specifically in response to the higher demand incongruent Stroop blocks.

#### Region-of-interest (ROI) analysis

To directly compare the magnitude of regional deactivation associated with the ‘rest *minus* task’ contrasts derived from both experiments an empirically-based region-of-interest (ROI) analysis was performed. Firstly, regions that demonstrated significant consistent deactivation (SPM voxel-level family-wise error rate corrected; *P* **_FWE_** *<*0.05; K_E_, ≥10 voxels) in one or more of the four ‘rest *minus* task’ contrasts were mapped via a conjunction analysis testing the global null hypothesis [31]. The global null approach was chosen as a means to characterize a common set of anatomical co-ordinates that would generally best represent the results of the four independent ‘rest *minus* task’ contrasts. For each of the regions identified, the volume-of-interest function in SPM5 was then used to estimate their relative magnitude of deactivation across these four contrasts. This function estimates the first eigenvariate of all voxel values contained within a user defined volume–in this instance, 5 mm radial spheres (sampling up to 81 voxels) centered on each regional cluster maxima identified from the conjunction analysis. For each respective region, magnitude data were then compared by one-way repeated-measures ANOVA with planned comparisons specifying ‘task contrast’ as the within-subject factor.

#### Performance-based analysis

To broadly examine the relationship between deactivation and task behavioral performance, a split-half comparison was performed dividing the sample on the basis of Stroop RT interference (ms) scores into high and low performance groups. We focused specifically on Stroop RT interference scores as they are assumed to index the “highest level” of task demand across both of our experiments. For this comparison, the groups were compared in terms of their corresponding pattern of deactivation associated with the **con-incon** contrast which estimated deactivation that was specific to the high demand incongruent task condition (SPM voxel-level, *P* **_uncorrected_** *<*0.001; K_E_, ≥10 voxels).

#### Supplementary functional connectivity analysis

To supplement the study's primary task-based analyses, an independent functional connectivity analysis of the DMN was also performed. For each subject, a four-minute continuous resting-state sequence was obtained immediately prior to the two task sequences with identical imaging acquisition parameters to that previously described. A single “seed-based” analysis of the ventral posterior cingulate cortex was performed in order to map the resting-state functional connectivity of the DMN: a conventional approach that has been adopted in numerous recent studies, e.g., [32], [33], [34]. Full details of this analysis are provided as Text S1.

## Results

### Task performance

Table 1 presents mean (± standard deviation, SD) RT scores and percentage error responses associated with both tasks.

**Table 1:** Behavioral Task Performance.

*Task*	Reaction Time, ms Mean (SD)	Errors, % Mean (SD)
**Emotional Face Matching Task**		
Shape matchingEmotional face matching	875.4 (229)1420.9 (383)*P*<0.0001	1.44 (2.1)2.52 (4.6)NS
**Self-Paced Stroop Task**		
Congruent trialsIncongruent trials	917.7 (305)1080.0 (353.2)*P*<0.0001	2.16 (2.2)3.26 (3.8)NS

SD, Standard deviation. NS, non-significant.

#### Task I

Subjects' RT scores to correctly answered trials were significantly slower during the face matching compared to shape matching blocks (*F*_1, 49_ = 274.2, *P*<0.0001), corresponding to a mean RT difference of 545.6 ms. Task accuracy did not differ between the two task conditions (*F*_1, 49_ = 2.26, *P* = 0.14) and was mostly at ceiling-levels (>95% accuracy).

#### Task II

Subjects' RT scores to correctly answered stimulus trials were significantly slower during the higher-demand incongruent blocks compared to congruent blocks (*F*_1, 49_ = 44.3, *P*<0.0001), corresponding to a mean Stroop RT interference effect (incongruent *minus* congruent RTs) of 162.2 milliseconds. Subjects' task accuracy was high overall (>95%) although they committed significantly more errors during the incongruent compared to congruent blocks (*F*_1, 49_ = 9.72, *P* = 0.003).

### Imaging results

**Figure 1:**
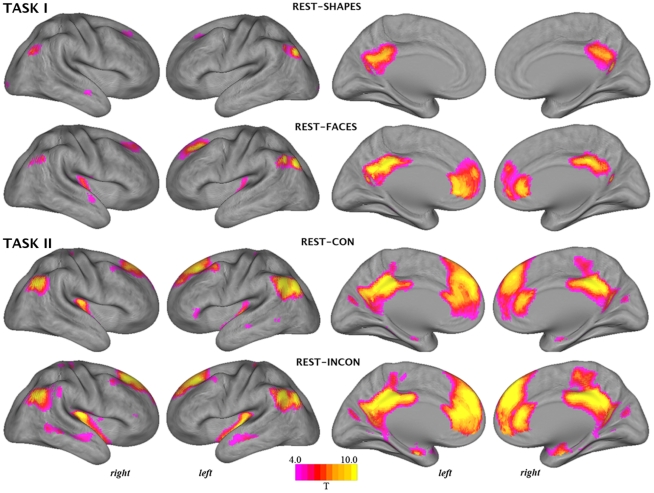
Whole-brain pattern of task-induced deactivations corresponding to the estimated ‘rest *minus* task’ contrasts.

**Figure 2:**
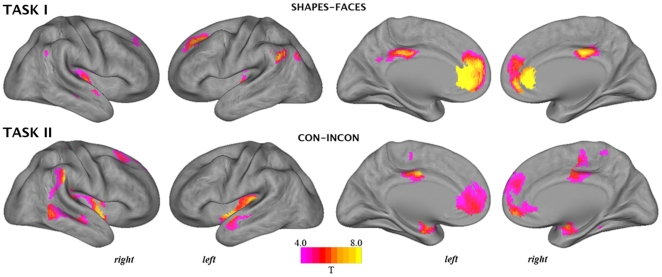
Whole-brain pattern of task-induced deactivations corresponding to the estimated ‘low demand *minus* high demand’ masked contrasts.

The patterns of task-induced deactivation from rest associated with both tasks are presented in Figure 1, whereas the patterns of deactivation specific to each task's high demand condition are presented in Figure 2. Imaging results are projected onto an inflated viewing of the cortical surface of the Population Average Landmark- and Surface-Based (PALS-B12) Atlas [35]. Tables 2 and 3 report all anatomical and statistical parametric results for tasks I and II, respectively.

**Table 2:** Task-Induced Deactivations Associated with Emotional Face Matching Task Performance.

Regions	Coordinates (x,y,z)			K_E_	T	BA
**REST-SHAPES**						
Angular gyrus	−44	−75	31	436	12.5	39
Angular gyrus	47	−70	31	334	11.1	39
Ventral posterior cingulate	10	−53	17	2604	10.12	31
Superior frontal cortex	−22	19	44	95	5.89	8
Lateral occipital cortex	26	−91	−6	11	5.34	18
Superior frontal cortex	21	22	50	42	5.03	8
**REST-FACES**						
Angular gyrus	−44	−76	31	716	16.67	39
Angular gyrus	50	−62	39	437	11.63	39
Ventral posterior cingulate	−11	−58	22	2959	11.43	31
Superior frontal cortex	−20	25	44	4304	11.30	8
Posterior insular cortex	38	−15	5	832	7.15	13
Superior frontal cortex	15	31	51	268	5.89	8
Posterior insular cortex	−40	−14	−3	190	5.74	13
Parahippocampal gyrus	−23	−33	−17	50	5.61	36
**SHAPES-FACES**						
Anterior cingulate cortex	−3	31	16	3641	10.33	10
Angular gyrus	−55	−60	28	363	8.77	39
Superior frontal cortex	−18	26	44	623	7.74	8
Mid cingulate cortex	2	−26	36	688	7.61	31
Supramarginal gyrus	54	−58	34	122	7.09	40
Posterior insular	38	−13	−2	1061	6.50	13
Posterior insular	−38	−11	−1	205	5.93	13
Middle temporal gyrus	55	−21	−15	12	5.02	21

**Coordinates (x, y, z)** are given in Talairach & Tournoux Atlas space (mm). Imaging co-ordinates were transformed from SPM-Montreal Neurological Institute (MNI) to Talairaich space using the icbm2tal transform (Lancaster et al. 2007) implemented in GingerALE (http://www.brainmap.org). Magnitude and extent statistics correspond to a minimum threshold of *P* **_FWE_** *<*0.05. **K_E_**, cluster size/number of contiguous voxels. **T**, SPM T-score statistics. **BA**, approximate Brodmann Area.

**Table 3:** Task-Induced Deactivations Associated with Self-Paced Stroop Task Performance.

Regions	Coordinates (x,y,z)			K_E_	T	BA
**REST-CON**						
Angular gyrus	−48	−73	27	1656	16.91	39
Angular gyrus	48	−67	33	1033	14.91	39
Dorsal posterior cingulate	−9	−43	38	4806	13.57	31
Dorsal medial frontal cortex	−18	24	50	8966	10.19	8
Posterior insular cortex	34	−18	16	633	9.74	13
Posterior insular cortex	−38	−16	1	314	7.74	13
Hippocampus-amygdala	20	−7	−19	31	6.08	27
Post-central gyrus	17	−35	57	79	6.06	4
Inferior frontal gyrus	−30	11	−15	29	5.90	47
Hippocampus-amygdala	−25	−9	−22	17	5.79	27
Superior frontal cortex	37	12	53	30	5.60	8
Post-central gyrus	−19	−37	60	13	5.38	5
**REST-INCON**						
Angular gyrus	−48	−73	27	1430	17.27	39
Dorsal posterior cingulate	−9	−41	36	6872	14.45	32
Angular gyrus	50	−65	31	1366	14.26	39
Ventral medial frontal cortex	−1	50	13	10155	12.23	10
Posterior insular cortex	32	−16	16	2664	11.87	13
Posterior insular cortex	−38	−16	1	1530	11.66	13
Hippocampus-amygdala	−27	−8	−24	112	9.05	27
Post-central gyrus	−19	−39	60	56	6.70	4
Middle temporal gyrus	58	−50	−1	62	6.07	37
**CON-INCON**						
Superior temporal gyrus	−49	−11	7	2136	8.62	22
Inferior parietal	56	−35	32	2529	7.99	40
Mid cingulate	−7	−17	42	590	7.44	31
Anterior cingulate cortex	1	48	−2	834	6.79	32
Middle temporal gyrus	53	−46	−3	215	6.33	21
Superior frontal gyrus	15	17	54	45	5.36	8

**Coordinates (x, y, z)** are given in Talairach & Tournoux Atlas space (mm). Imaging co-ordinates were transformed from SPM-Montreal Neurological Institute (MNI) to Talairaich space using the icbm2tal transform (Lancaster et al. 2007) implemented in GingerALE (http://www.brainmap.org). Magnitude and extent statistics correspond to a minimum threshold of *P* **_FWE_** *<*0.05. **K_E_**, cluster size/number of contiguous voxels. **T**, SPM T-score statistics. **BA**, approximate Brodmann Area.

#### Task I

For the **rest-shapes** and **rest-faces** task contrasts (Fig. 1), there was apparent overlapping deactivation of posterior cingulate, inferior parietal (angular gyrus) and superior frontal components of the DMN under both levels of task demand, but further distinct deactivation associated with the higher demand face matching blocks. Figure 2 presents those regions that deactivated specifically in response to the face matching compared to shape matching blocks (**shapes-faces** contrast). Regions that showed greater deactivation under the higher demand task condition included the dorsal posterior cingulate cortex, rostral anterior cingulate extending to ventral medial frontal cortex, superior frontal cortex, supramarginal gyrus, middle temporal gyrus and the posterior insular cortex.

#### Task II

For the **rest-con** and **rest-incon** task contrasts (Fig. 1), there was apparent overlapping deactivation of posterior cingulate, angular gyrus, medial frontal and superior frontal components of the DMN and the posterior insular cortex under both levels of task demand, but further distinct deactivation associated with the higher demand incongruent task blocks. Figure 2 presents those regions that deactivated specifically in response to the incongruent compared to congruent Stroop blocks (**con-incon** contrast), which included the dorsal posterior cingulate extending to mid cingulate cortex, posterior insular extending to mid insular cortex, the supramarginal gyrus, middle and superior temporal gyrus, rostral anterior cingulate extending to ventral medial frontal cortex, and extended amygdala-hippocampal complex.

#### Region-of-interest (ROI) analysis

**Figure 3:**
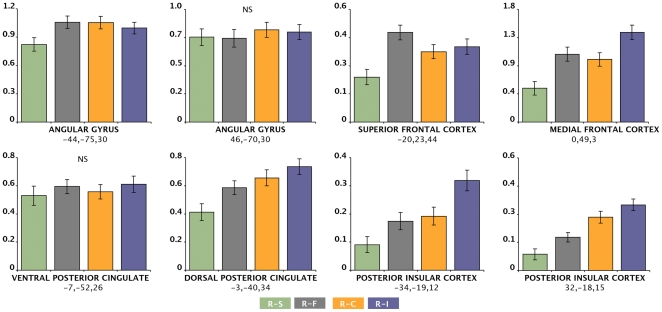
Region-of-interest analysis of the corresponding magnitude of deactivations from rest observed across both tasks. **r–s**, rest *minus* shape matching; **r–f**, rest *minus* face matching; **r–c**, rest *minus* congruent trials; **r–i**, rest *minus* incongruent trials. **NS** = no significant main effect of condition. Y-axis values corresponding to estimated SPM contrast β values (mean ± SEM). Regional activity co-ordinates are provided in Talairach & Tournoux Atlas space (x, y, z mm).

Figure 3 presents the relative magnitude of deactivation of those brain regions demonstrating consistent deactivation across the four ‘rest *minus* task’ contrasts: **rest-shapes, rest-faces** (Task I; face matching task), **rest-con, rest-incon** (Task II, Stroop task). Significant main effects of task contrast were observed for the left angular gyrus (F_3,147_ = 5.3, *P*<0.002), superior frontal cortex (F_3,147_ = 9.5, *P*<0.001), ventral medial frontal cortex (F_3,147_ = 15.6, P<0.001), dorsal posterior cingulate cortex (F_3,147_ = 12.2, *P*<0.01), left posterior insular cortex (F_3,147_ = 14.7, *P*<0.001), and right posterior insular cortex (F_3,147_ = 14.7, *P*<0.001).

The results of planned statistical contrasts can be summarized as follows: for regions demonstrating a significant main effect of condition, there was more pronounced deactivation observed in all contrasts compared to the **rest-shapes** contrast (all *P* values<0.01). For the superior frontal cortex, there was more pronounced deactivation observed in the **rest-faces** compared to **rest-con** contrasts (*P*<0.05). For the ventral medial frontal cortex there was more pronounced deactivation observed in the **rest-incon** compared to **rest-faces** contrasts (*P*<0.01) and **rest-con** (*P*<0.001) contrasts. For the dorsal posterior cingulate cortex there was similarly more pronounced deactivation observed in the **rest-incon** compared to **rest-faces** contrasts (*P*<0.01) and **rest-con** (*P*<0.001) contrasts. The right posterior insular cortex demonstrated a stepwise increase in deactivation between the **rest-shapes, rest-faces, rest-con** and **rest-incon** contrasts (all *P* values <0.01).

#### Performance-based analysis

**Figure 4:**
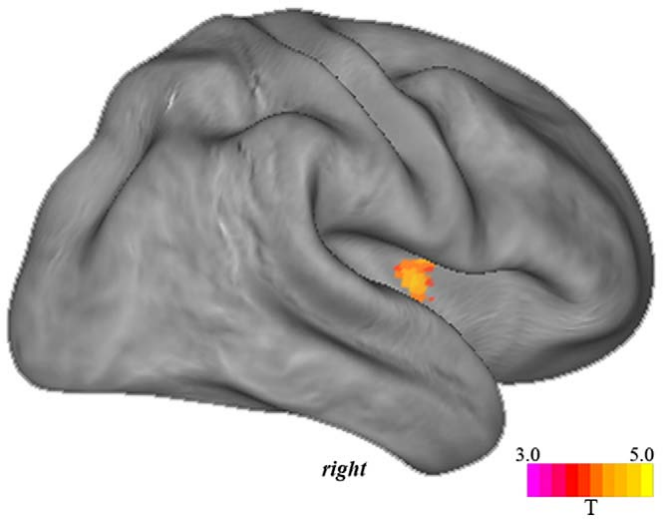
The region of right posterior-to-mid insular cortex deactivation that discriminated low from high performing subjects on the Stroop task.

When comparing groups based on a group split-half analysis of Stroop RT interference, no regions of greater deactivation were found to distinguish the high from low performing groups. By comparison, low performing subjects were found to demonstrate significantly greater deactivation of the right posterior-to-mid insular cortex (*x, y, z* = 42, −2, 8; T = 3.79). Results of this performance-based analysis of task difficulty are presented in Figure 4.

#### Supplementary functional connectivity analysis

**Figure 5:**
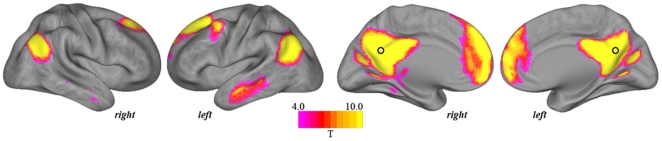
Resting-state functional connectivity of the default mode network. Black circles highlight the approximate anatomical placement of the posterior cingulate cortex ‘seed’ regions of interest that were defined for this analysis.

Using the ventral posterior cingulate cortex as a reference ‘seed’, the characteristic anatomy of the DMN was additionally mapped under independent resting-state conditions to provide a qualitative comparison with the primary task-based findings. In addition to posterior medial wall regions, a significant pattern of functional connectivity was defined between the seed region and the medial and superior frontal cortex, bilateral angular gyri, and left inferior temporal cortex (*P* **_FWE_**<0.05; K_E_, ≥10 voxels). Figure 5 presents the results of this supplementary resting-state analysis. The corresponding anatomical and statistical parametric results are reported in Supporting Information Table S1.

## Discussion

Our results can be summarized as two primary observations: Firstly, supporting initial predictions, increases in task demand were found to consistently modulate the regional anatomy of DMN deactivation. This observation was perhaps most apparent for the posterior cingulate cortex, whose deactivation extended anterior to the mid-cingulate cortex with increases in task demand. Secondly, increased task demand led to additional deactivation of “non-DMN regions”, most notably, the posterior insular and surrounding cortex. Both observations appeared to generalize well across the distinct task scenarios. As additional findings, deactivation of the posterior-to-mid insular cortex was found discriminate low from high performing subjects on the Stroop task, further supporting a link between this region and experienced task demand, while a supplementary functional connectivity analysis did not implicate the posterior insular cortex as part of the default mode network under resting-state conditions.

Only a marginal degree of selectivity was observed between the two tasks and their corresponding patterns of deactivation. As highlighted in Figure 1, performance of the Stroop task, overall, led to more pronounced deactivation when compared to the face matching task. This difference is likely to reflect the added cognitive demand associated with this task (i.e., fast self-paced responding), which presumably enhances deactivations by minimizing the potential occurrence of task-independent thoughts during task blocks [17], although this was not specifically evaluated. A second difference between the tasks was observed with regards to the deactivation of the amygdala-hippocampal region. These regions are robustly activated by the face matching task together with other characteristic regions (e.g., fusiform gyrus; [28]), and as such were not expected to show significant deactivation. While this expectation was met, these regions were found to robustly deactivate during Stroop task performance, particularly at high task demand levels. This finding would appear to depend on the specific characteristics of this task version, as the deactivation of these regions has not typically been observed with other related ‘high level’ cognitive tasks [4], [5], [8].

Accounting for such differences, the anatomy of deactivations was mostly overlapping between the two tasks and across the distinct contrasts. Figure 1 highlights all deactivations from rest, which globally reproduced the characteristic anatomy of the DMN. Figure 2 highlights the brain regions that demonstrated a specific increase in deactivation when contrasting each task's low and high demand conditions to map the specific correlates of increased task demand. This contrast revealed a distinct pattern of deactivation encompassing the dorsal posterior cingulate cortex extending to mid-cingulate cortex, the ventral medial frontal cortex and the posterior insular and surrounding cortex. While the deactivation of DMN regions has been linked to task demands in past studies [21], [22], [36], our results indicate that such modulation influences the precise anatomy of regional deactivations, as seen with the posterior cingulate cortex, and extends to non-DMN regions, particularly, the posterior insular cortex.

While deactivations of the dorsal posterior to mid cingulate cortex and ventral medial frontal cortex would be considered to represent the DMN as currently conceptualized, the same cannot be said for the posterior insular cortex. This distinction is perhaps best highlighted by resting-state functional connectivity studies, which have become a conventional approach for mapping the functional anatomy of the DMN. These studies, when either adopting a region-of-interest or whole brain approach, do not implicate the posterior insular cortex as a common functional component the DMN [5], [17], [32], [33], [37]. We confirmed this observation in the current work by performing an independent resting-state analysis of the DMN centered on the posterior cingulate cortex, which failed to define significant functional connectivity with the posterior insular or surrounding cortex (Fig. 5). This finding is in close agreement with recent detailed studies of posterior insular cortex functional connectivity based on resting-state fMRI [38], [39], [40].

In addition to its stronger tendency to deactivate at high task demand levels, the posterior insular cortex, compared to posterior DMN regions, displayed less tendency to deactivate at lower levels of task demand (Fig. 3.), suggesting that it may be less readily responsive to changes in external demands than what has been indicated for some DMN regions [41]. It is interesting to consider evidence which suggests that “background” or baseline activity at the level of the posterior insular cortex may be set particularly high as a result of its continuous multisensory inputs [42]. This notion has been invoked to explain the absence of SII/posterior insular responses to low and mild intensity nociceptive stimulation, but marked responses to high intensity stimulation, compared to other pain matrix regions [42], [43], [44]. If true, such a characteristic could explain its apparent tendency to activate and deactivate at only relatively high stimulation thresholds.

Whereas the DMN has been linked to higher-order aspects of self-related cognition, the posterior insular cortex is thought to contribute to integrated representations of bodily afferent processes that underlie the subjective experience of body-ownership, agency, and feeling-states [45], [46], [47]. Therefore, one hypothesis may be that functional deactivations of this brain region correspond to some degree of reduced self-awareness at the level of integrated sensory and interoceptive processing. As distinct from other DMN regions, posterior insular deactivation appears mostly during states of high attentional allocation to the environment (e.g., intense concentration, vigilance, effort), which may unmask a baseline level of activity that is specific to this region. This hypothesis would be generally consistent with other evidence linking decreased awareness of somatosensory and nociceptive stimulation to reduced posterior insular activity, among other areas, during states of high attentional demand or cognitive distraction [26], [48]. For example, thermal pain-related activation of posterior insular cortex and subjective pain intensity is diminished by Stroop task performance [26].

Although speculative, the current results taken with other recent evidence raise the interesting possibility that task-induced deactivations from resting baselines emerge in a graded and organized manner that may reflect the progressive detachment of different elements of conscious self-awareness and perceptual processes [21], [22], [41]. We have made a distinction between the ‘earlier’ deactivation of posterior DMN regions often suggested to underlie elements of egocentric self-awareness, mental imagery, and associated mnemonic processes, versus the ‘later’ deactivation of the posterior insular cortex and the awareness of integrated bodily afferent processes. A further intermediate step may be represented in the deactivation of ventral medial frontal and medial temporal lobe structures, which have been broadly linked to self-referential processes cutting across volitional, emotional and motivational domains [49], [50]. However, further studies will be necessary to advance such ideas, given that our experiments were not designed to modulate deactivations across multiple graded levels of task difficulty, or to assess different aspects of self-awareness during the rest and task periods. It will also be relevant for studies to examine the influence of regions, such as the anterior insular cortex, in modulating task-induced deactivations, given further observations described in recent work [51].

An additional caveat to this work or indeed any fMRI study examining task-induced deactivations relates to the unconstrained influence that the scanning environment might play in modulating resting baseline activity. Factors such as scanner noise, MRI-related subject anxiety and physical discomfort may have the effect of promoting (or even reducing) observed deactivations by representing salient sources of stimulation, particularly during off-task imaging periods. While not likely to account for the deactivation phenomena observed in functional imaging studies, they may interact to define a specific reference state that is qualitatively distinct from resting activity measured with other neuroimaging techniques [42]. These limitations considered, the current work nonetheless indicates that further studies of task-induced deactivations may be relevant for advancing current perspectives of brain function related to the DMN. Taken together, our findings argue that such deactivations are more broadly encoded that what is presently thought.

## Supporting Information

Table S1 **Resting-State Functional Connectivity of the Posterior Cingulate Cortex.** **Coordinates (x, y, z)** are given in Talairach & Tournoux Atlas space (mm). Imaging co-ordinates were transformed from SPM-Montreal Neurological Institute (MNI) to Talairaich space using the icbm2tal transform (Lancaster et al. 2007) implemented in GingerALE (http://www.brainmap.org). Magnitude and extent statistics correspond to a minimum threshold of *P* **_FWE_** *<*0.05. **K_E_**, cluster size/number of contiguous voxels. **T**, SPM T-score statistics. **BA**, approximate Brodmann Area.
(DOC)

Text S1 **Supplementary functional connectivity analysis.**
(DOC)
