# Supplementary material for: Task-Induced Deactivation from Rest Extends beyond the Default Mode Brain Network
Source: PLoS One. 2011 Jul 29;6(7):e22964. doi: 10.1371/journal.pone.0022964 (PMC3146521; doi:10.1371/journal.pone.0022964)
Supplement: Table S1 — Resting-State Functional Connectivity of the Posterior Cingulate Cortex. Coordinates (x, y, z) are given in Talairach & Tournoux Atlas space (mm). Imaging co-ordinates were transformed from SPM-Montreal Neurological Institute (MNI) to Talairaich space using the icbm2tal transform (Lancaster et al. 2007) implemented in GingerALE (http://www.brainmap.org). Magnitude and extent statistics correspond to a minimum threshold of P FWE <0.05. KE, cluster size/number of contiguous voxels. T, SPM T-score statistics. BA, approximate Brodmann Area. (DOC) [file pone.0022964.s001.doc]

**Table S1**. Resting-State Functional Connectivity of the Posterior Cingulate Cortex

| **Regions** | **Coordinates**  **(x, y, z)** | | | **K_E_** | **T** | **BA** |
| --- | --- | --- | --- | --- | --- | --- |
| Ventral posterior cingulate | -7 | -52 | 23 | 9087 | 36.98 | 39 |
| Angular gyrus | 45 | -64 | 24 | 1875 | 20.00 | 39 |
| Angular gyrus | -45 | -69 | 26 | 2727 | 19.86 | 31 |
| Medial frontal cortex | -1 | 56 | 6 | 8338 | 14.37 | 8 |
| Inferior temporal cortex | -54 | -5 | -19 | 469 | 9.53 | 13 |

**Coordinates (x, y, z)** are given in Talairach & Tournoux Atlas space (mm). Imaging co-ordinates were transformed from SPM-Montreal Neurological Institute (MNI) to Talairaich space using the icbm2tal transform (Lancaster et al. 2007) implemented in GingerALE ([http://www.brainmap.org](http://www.brainmap.org/)). Magnitude and extent statistics correspond to a minimum threshold of *P***_FWE_** *<* 0.05. **K_E_**, cluster size / number of contiguous voxels. **T**, SPM T-score statistics. **BA**, approximate Brodmann Area.
