# Supplementary material for: Task-Induced Deactivation from Rest Extends beyond the Default Mode Brain Network
Source: PLoS One. 2011 Jul 29;6(7):e22964. doi: 10.1371/journal.pone.0022964 (PMC3146521; doi:10.1371/journal.pone.0022964)
Supplement: Text S1 — Supplementary functional connectivity analysis. (DOC) [file pone.0022964.s002.doc]

*Supplementary functional connectivity analysis*

To map the functional connectivity of the DMN under resting-state conditions, a seed-based functional connectivity analysis of the ventral posterior cingulate cortex was performed. The anatomical location of our posterior cingulate cortex seed region was based on the findings of Harrison et al. (2008), which independently assessed DMN functional connectivity under resting-state conditions using a model free analysis approach. The precise seed region was defined as a 5-mm radial sphere at the following co-ordinates x=-3, y=54, z=30. Functional time-series were then extracted subject-wise for this seed region by calculating the mean voxel value across the time-series using the MarsBaR ROI toolbox in MNI stereotaxic space. In addition to the posterior cingulate seed, we derived estimates of white matter (WM), cerebrospinal fluid (CSF), and global brain signal fluctuations to include as regressors of no interest (“nuisance variables”) in the resting time-series analyses. To do so, MNI-space template segments of grey matter, WM and CSF were thresholded at 70% tissue probability type and binarized to create nuisance variable masks, together with a binary mask of the global brain volume (summed from GM, WM, and CSF segments). Nuisance signals were then extracted for each mask respectively by calculating the mean voxel value across the time-series. These nuisance signals are typically adjusted for resting-state (i.e., continuous) functional connectivity studies as they reflect global signal fluctuations of non-neuronal origin (e.g., physiological artifacts associated with variables such as cardiac and respiratory cycles, CSF motion, and scanner drift).

Functional connectivity maps were estimated for the DMN by including the posterior cingulate seed and nuisance signals as predictors of interest/no-interest in a first-level (subject-wise) general linear model analysis. A high-pass filter set at 128 seconds was used to remove low frequency drifts below ~ 0.008 Hz. Prior to model estimation, each of the three nuisance covariates were orthogonalized (using an iterative Gram-Schmidt method) and then removed from the posterior cingulate time-series by linear regression, resulting in a general linear model that comprised a ‘noise-cleaned’ posterior cingulate seed and three orthogonal nuisance variables. Contrast images were generated for each subject by estimating the regression coefficient between all brain voxels and the posterior cingulate time-series. These images were then included in a group random-effects analyses (one sample t-test; SPM family-wise error rate corrected; *P*_FWE_ < 0.05, _KE_, ≥ 10 voxels).

**Reference**

Harrison BJ, Pujol J, Lopez-Sola M, Hernandez-Ribas R, Deus J, Ortiz H, Soriano-Mas C, Yucel M, Pantelis C, Cardoner N. (2008): Consistency and functional specialization in the default mode brain network. Proc Natl Acad Sci U S A 105(28):9781-6.
